# Novel stereocontrolled syntheses of tashiromine and epitashiromine

**DOI:** 10.3762/bjoc.11.66

**Published:** 2015-04-30

**Authors:** Loránd Kiss, Enikő Forró, Ferenc Fülöp

**Affiliations:** 1 Institute of Pharmaceutical Chemistry, University of Szeged, H-6720 Szeged, Eötvös u. 6, Hungary; 2 Stereochemistry Research Group of the Hungarian Academy of Sciences, University of Szeged, H-6720 Szeged, Eötvös u. 6, Hungary

**Keywords:** alkaloids, amino acids, ring closure, ring opening, stereocontrolled synthesis

## Abstract

A novel stereocontrolled approach has been developed for the syntheses of tashiromine and epitashiromine alkaloids from cyclooctene β-amino acids. The synthetic concept is based on the azetidinone opening of a bicyclic β-lactam, followed by oxidative ring opening through ring C–C double bond and reductive ring-closure reactions of the *cis*- or *trans-*cyclooctene β-amino acids.

## Introduction

**Figure 1:**
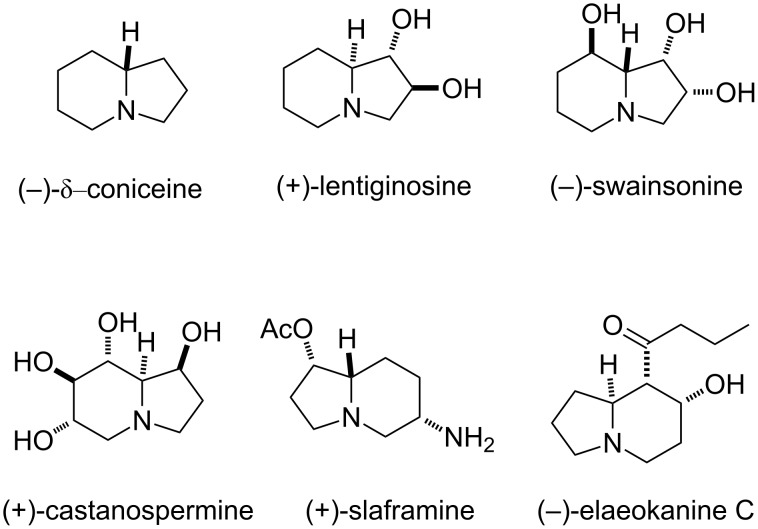
Some indolizidine alkaloids.

Indolizidine alkaloids are an important class of naturally occurring compounds which have received considerable attention as a result of their valuable physiological properties. A number of representatives of this class exhibit glycosidase inhibitory activity or antimetastatic, anticancer, antitumour or anti-HIV properties [1–3]. A large number of natural products contain an indolizidine framework, among them (−)-δ-coniceine, (−)-swainsonine, indolizidine 167B [4–10], (+)-lentiginosine [11–15], (+)-slaframine [16], (−)-elaeokanine C [17], (+)-cyclizidine [18], lepadiformine [19], the highly oxygenated (+)-castanospermine [20–21], or pumiliotoxin [22]. Figure 1 illustrates the structures of several such compounds.

**Figure 2:**
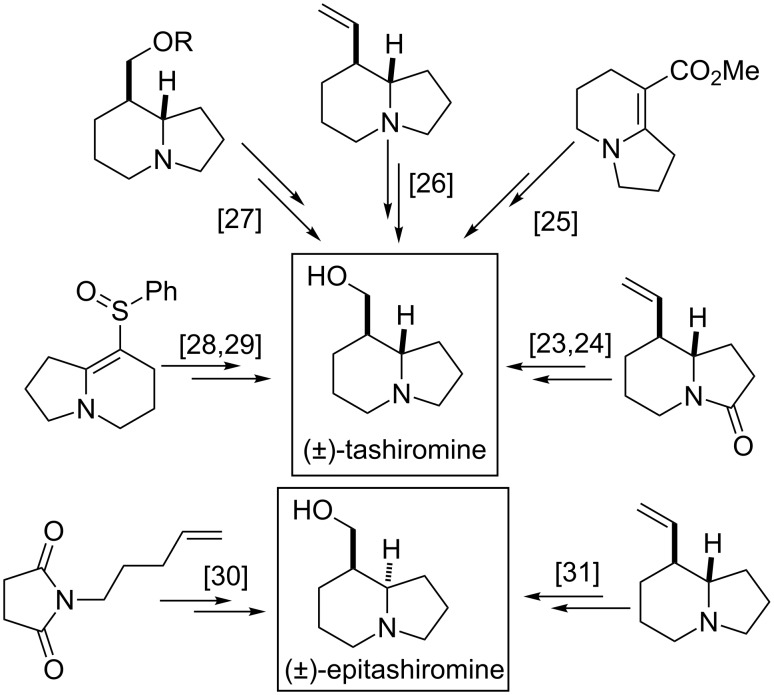
Approaches to racemic tashiromine and epitashiromine.

Tashiromine is a natural indolizidine alkaloid isolated from *Maackia tashiroi* (1990). Strategies for the synthesis of indolizidine derivatives have received considerable interest from synthetic and medicinal chemists (Figure 2). A number of synthetic approaches have been described earlier for construction of the indolizidine framework; access to tashiromine in racemic form can be achieved through the alkylation of succinimide, followed by ring closure via acyliminium intermediates [23–24], the reduction of cyclized pyridinium salts [25], iminium cascade cyclization [26], alkyne-mediated hydroformylation–cyclization [27], or electrophilic pyrolidinone alkylation followed by ring closure [28–29]. Pyrrolidine alkylation and nucleophilic ring closure followed by C–C double bond hydroboration [30] leads to racemic epitashiromine, as does the *N*-alkylated succinimide transformation through the corresponding indolizidinone [31].

**Figure 3:**
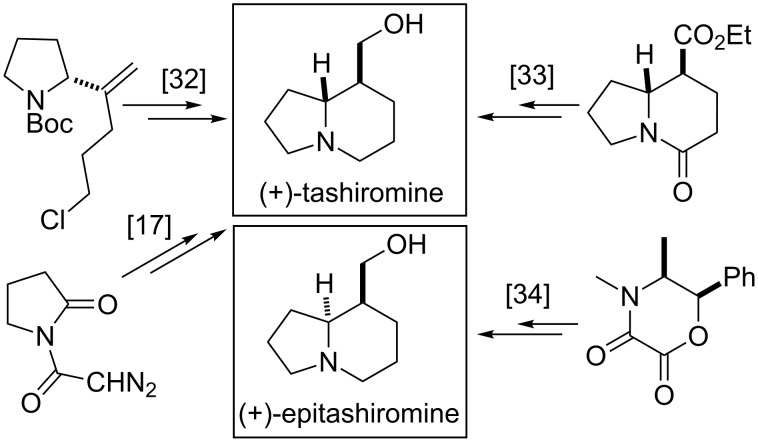
Synthetic routes to (+)-tashiromine and (+)-epitashiromine.

**Figure 4:**
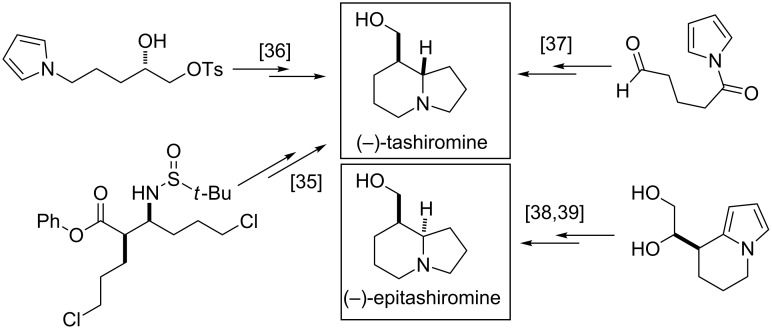
Synthetic routes to (−)-tashiromine and (−)-epitashiromine.

Several synthetic procedures have also been developed for the preparation of tashiromine or epitashiromine enantiomers.

(+)-Tashiromine has been synthetized from a pyrrolidinone derivative through chiral Lewis acid-catalysed cyclization to substituted pyrrolidinones [17], by the intramolecular cyclization of a chiral alkenylated pyrrolidinone, followed by hydroxylation [32], or by the intramolecular ring closure of chiral pyrrolidine diesters followed by ester and oxo group reduction [33], while the syntheses of (+)-epitashiromine starts from a chiral morpholine derivative, with nitrone 1,3-dipolar cycloaddition and reduction [34] (Figure 3).

(−)-Tashiromine has been accessed through the ring closure of difunctionalized acyclic chiral sulfonamide-based β-amino acids [35], the cyclization of pyrrole derivatives with a chiral side-chain [36], or the enantioselective arylation of pyrrole, followed by saturation [37]. The transformation of chiral functionalized pyrrole or pyrrolidine derivatives has served as the basis of the construction of (−)-epitashiromine [38–39] (Figure 4).

**Figure 5:**
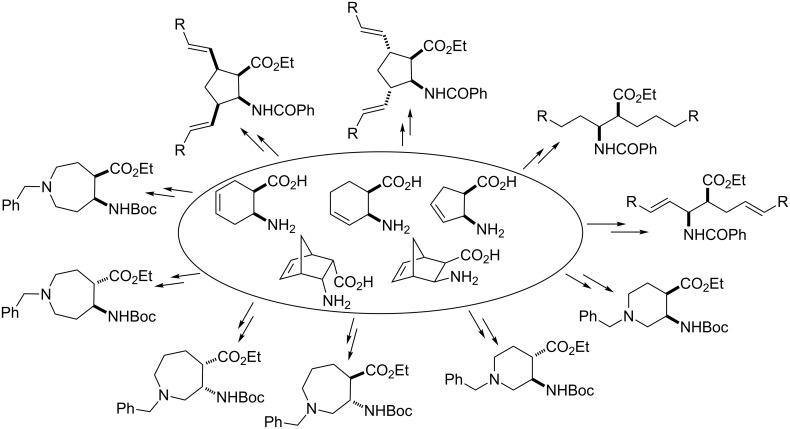
Oxidative functionalizations of cyclic β-amino acids.

The oxidative functionalization of cyclic β-amino acid derivatives has been reported to be a convenient route for the preparation of *N*-heterocyclic β-amino acid derivatives [40–41] or for the stereocontrolled synthesis of functionalized cispentacins [42] and their acyclic counterparts [43–44] (Figure 5). The oxidative ring cleavage of various vicinal diols and the transformation of the resulting dialdehyde intermediates has been efficiently applied in recent years for the synthesis of a series of valuable organic molecules [45–52]. In particular, Davies and co-workers have utilized the oxidative ring opening of cyclic vicinal diols followed by ring closure for access to pyrrolizidine alkaloids [45].

## Results and Discussion

**Scheme 1:**
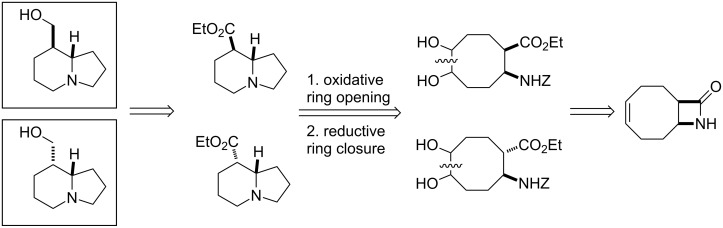
Retrosynthesis of tashiromine and epitashiromine.

We describe here a novel access route for the synthesis of tashiromine and epitashiromine by starting from an unsaturated bicyclic β-lactam. The retrosynthetic concept of the synthesis is represented on Scheme 1 and was based on the lactam ring opening, in continuation followed by oxidative ring opening of the formed β-amino esters and by reductive ring closure as key steps.

**Scheme 2:**
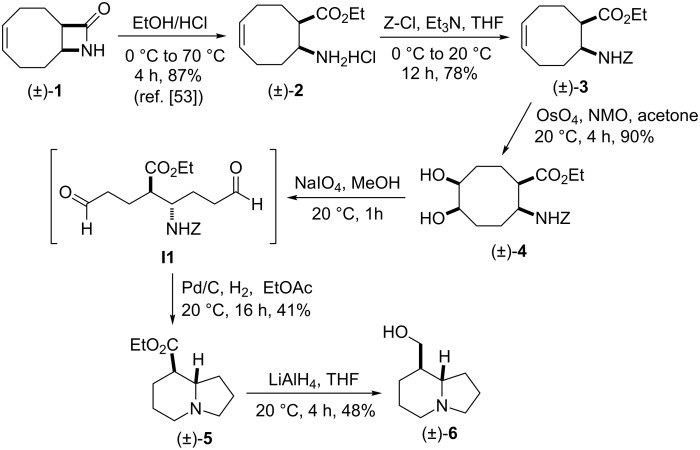
Synthesis of (±)-tashiromine ((±)-**6**).

Bicyclic β-lactam (±)-**1** [53–54] was first transformed by azetidinone opening to the corresponding amino ester hydrochloride (±)-**2** [53–54], *N*-protection of which with benzyl chloroformate (Z-Cl) afforded protected amino ester (±)-**3** in 78% yield. In agreement with our earlier observations [40–42] C–C double bond functionalization of the cyclooctene β-amino ester via dihydroxylation with *N*-methyl morpholine *N*-oxide (NMO) in the presence of OsO_4_ afforded the corresponding *all-cis* dihydroxylated ethyl β-aminocyclooctanecarboxylate (±)-**4** in 90% yield (for dihydroxylation, see also reference [54]) (Scheme 2). Amino ester (±)-**4** was next subjected through its vicinal diol moiety to oxidative ring opening with NaIO_4_ in MeOH at 20 °C, which resulted (monitored by TLC) in the corresponding ring-opened unstable diformyl intermediate (**I1**), which after work-up was immediately used without further purification (for several similar types of acyclic diformyl intermediates, see references [40–41,43–44]). Thus, the crude material was submitted to catalytic hydrogenolysis and after *N*-deprotection underwent double cyclization–reduction to furnish indolizidine ester (±)-**5** in 41% yield after purification by chromatography.

Reduction of the ester group of (±)-**5** with an excess of LiAlH_4_ in THF at 20 °C gave the corresponding tashiromine (±)-**6** [35–37], which was isolated in 48% yield after purification by column chromatography (Scheme 2). The stereochemistry of (±)-**6** was unequivocally assigned by NMR data, which were consistent with those reported [35–37].

A similar strategy was applied for the synthesis of epitashiromine. On reaction with NaOEt in EtOH at 20 °C, ethyl *cis*-β-aminocyclooctenecarboxylate (±)-**3** underwent epimerization at C-1, leading after 18 h to an equilibrium mixture of *cis* and *trans* amino esters (1:1 ratio determined by ^1^H NMR on the crude mixture), the required *trans* isomer (±)-**7** being separated from the *cis* counterpart and isolated in a yield of 48% by means of column chromatography. Dihydroxylation of (±)-**7** with NMO/OsO_4_ next afforded an oily mixture of *cis* and *trans* dihydroxylated cyclooctane β-amino esters (diastereomeric mixture of (±)-**8**) in 77% overall yield after column chromatography. Our attempts to separate this nearly 1:1 mixture of the two dihydroxylated stereoisomers (determined on the basis of ^1^H NMR data) failed, but the mixture could be applied in the next ring-opening oxidation step, since it gave only one open-chain diformyl intermediate **I2**.

**Scheme 3:**
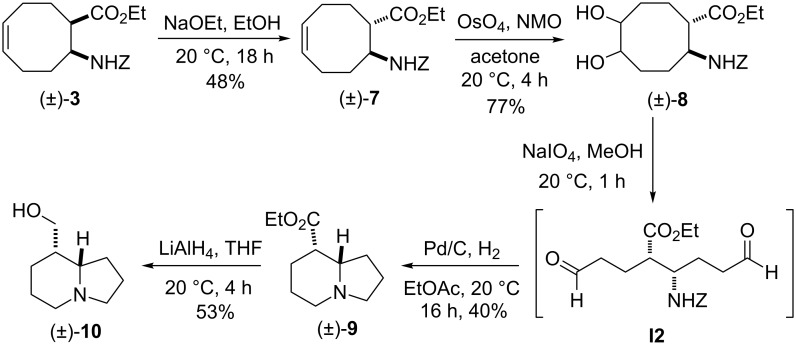
Synthesis of (±)-epitashiromine ((±)-**10**).

Similarly to the *cis* isomer, this unstable dialdehyde intermediate was subjected without isolation to catalytic hydrogenolysis, followed by reductive cyclization, to give the corresponding indolizidine ester (±)-**9** in 40% yield. Finally, ester reduction with LiAlH_4_ in THF resulted in epitashiromine (±)-**10** [32,34,39] in 53% yield after isolation by chromatography (Scheme 3). The stereochemistry of (±)-epitashiromine was assigned by NMR data, which were in agreement with those reported [32,34,39].

## Conclusion

In summary, a novel stereocontrolled efficient method has been presented for the synthesis of tashiromine and epitashiromine alkaloids in six or seven steps, based on the preparation of *cis* or *trans* cyclooctene β-amino esters, followed by their oxidative ring cleavage and double reductive ring-closure reactions.

## Experimental

### General procedure for the Z-protection of amino esters

To a solution of amino ester hydrochloride ((±)-**2** or (−)-**2**) [29] (17.8 mmol) in THF (40 mL), Et_3_N (9 mL) was added at 0 °C, followed by 7.8 mL (1 equivalent) of Z-Cl (a 50% solution in toluene). The mixture was stirred for 14 h at 20 °C, and then was diluted with EtOAc (120 mL). The organic layer was washed with H_2_O (3 × 60 mL), dried (Na_2_SO_4_), and concentrated under reduced pressure. The crude material was purified by column chromatography on silica gel (*n*-hexane/EtOAc 4:1), affording the amino ester.

### General procedure for the dihydroxylation of amino esters

To a solution of *cis* or *trans* Z-protected amino ester ((±)-**3**, (−)-**3** or (±)-**7**) (2.9 mmol) in acetone (30 mL) and H_2_O (1 mL), NMO (1.5 equivalents) and 2% OsO_4_ in *t*-BuOH (0.7 mL) were added and the mixture was stirred at 20 °C for 4 h. A saturated aqueous solution of Na_2_SO_3_ (40 mL) was then added, the mixture was extracted with CH_2_Cl_2_ (3 × 30 mL), and the organic layer was dried (Na_2_SO_4_) and concentrated under reduced pressure. The crude product was purified by column chromatography on silica gel (*n*-hexane/EtOAc 1:4) to give the dihydroxylated amino ester.

### General procedure for epimerization of the *cis*-amino ester

To a solution of *cis N*-protected amino ester ((±)-**3** or (−)-**3**) (3.3 mmol) in EtOH (30 mL), EtONa (1.5 equivalents) was added at 0 °C and the mixture was stirred at 20 °C for 18 h. H_2_O (70 mL) was then added, the mixture was extracted with CH_2_Cl_2_ (3 × 30 mL), and the organic layer was dried over Na_2_SO_4_ and concentrated under reduced pressure. The crude material was purified by column chromatography on silica gel (*n*-hexane/EtOAc 9:1) to give the *trans* isomer as a colourless oil.

### General procedure for the oxidative ring opening/reductive ring closure of dihydroxylated amino esters

To a solution of dihydroxylated amino ester ((±)-**4**, (±)-**8** or (−)-**8**) (2.46 mmol) in MeOH (25 mL), NaIO_4_ (2 equivalents) was added and the mixture was stirred at 20 °C for 45 min. It was then diluted with H_2_O (50 mL) and extracted with CH_2_Cl_2_ (3 × 20 mL). The organic layer was dried (Na_2_SO_4_) and concentrated under reduced pressure. The crude mixture was dissolved in EtOAc (30 mL), Pd/C (150 mg) was added and the mixture was stirred at 20 °C for 16 h. The catalyst was next filtered off through Celite. The crude mixture was then purified by column chromatography on silica gel (CH_2_Cl_2_/MeOH 95:5 or CH_2_Cl_2_/MeOH 9:1) to give the indolizidine derivative.

### General procedure for reduction of the ester

To a solution of indolizidine carboxylate ((±)-**5**, (±)-**9** or (−)-**9**) (1 mmol) in dry THF (15 mL), LiAlH_4_ (5 equivalents) was added at 0 °C and the mixture was stirred at 20 °C for 4 h. It was then cooled to 0 °C, H_2_O (2 mL) was added dropwise and the solid formed was filtered off through Celite. The filtrate was extracted with CH_2_Cl_2_ (3 × 15 mL), and the combined organic layers were dried (Na_2_SO_4_) and concentrated under reduced pressure. The crude oil was purified by column chromatography on silica gel (CH_2_Cl_2_/MeOH/NH_4_OH 90:8:2 or CH_2_Cl_2_/MeOH/NH_4_OH 90:5:5) to give the alkaloid.

Ethyl (1R*,2S*)-2-(benzyloxycarbonylamino)cyclooct-5-enecarboxylate ((±)-3)


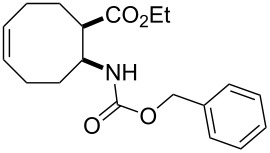


A colourless oil (*R*_f_ 0.6, *n*-hexane/EtOAc 4:1); yield: 78%; ^1^H NMR (400 MHz, CDCl_3_) δ 1.28 (t, *J* = 7.1 Hz, 3H, CH_3_), 1.76–1.87 (m, 2H, CH_2_), 1.88–1.97 (m, 1H, CH_2_), 1.98–2.07 (m, 1H, CH_2_), 2.09–2.18 (m, 2H, CH_2_), 2.22–2.31 (m, 1H, CH_2_), 2.43–2.52 (m, 1H, CH_2_), 2.82–2.89 (m, 1H, H-1), 4.12–4.21 (m, 2H, OCH_2_), 4.22–4.30 (m, 1H, H-2), 5.08 (s, 2H, OCH_2_), 5.30 (brs, 1H, N-H), 5.60–5.74 (m, 2H, H-5 and H-6), 7.33–7.48 (m, 5H, Ar-H); ^13^C NMR (100 MHz, DMSO) δ 14.9, 24.4, 25.1, 26.6, 32.3, 46.4, 52.1, 60.6, 66.1, 128.5, 128.6, 129.2, 129.5, 130.7, 138.0, 156.3, 174.5; anal. calcd for C_19_H_25_NO_4_: C, 68.86; H, 7.60; N, 4.23; found: C, 68.59; H, 7.31; N, 3.93.

Ethyl (1R*,2S*,5R*,6S*)-2-(benzyloxycarbonylamino)-5,6-dihydroxycyclooctanecarboxylate ((±)-4)


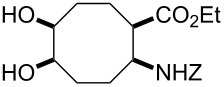


A colourless oil (*R*_f_ 0.4, *n*-hexane/EtOAc 1:4); yield: 90%; ^1^H NMR (400 MHz, CDCl_3_) δ 1.26 (t, *J* = 7.15 Hz, 3H, CH_3_), 1.62–1.94 (m, 4H, CH_2_), 1.97–2.28 (m, 4H, CH_2_), 2.77–2.82 (m, 1H, H-1), 3.83–3.89 (m, 2H, H-5 and H-6), 4.02–4.10 (m, 1H, H-2), 4.11–4.19 (m, 2H, OCH_2_), 5.09 (m, 2H, OCH_2_), 5.49 (brs, 1H, N-H), 7.36–7.48 (m, 5H, Ar-H); ^13^C NMR (100 MHz, DMSO) δ 14.8, 21.2, 26.9, 28.5, 29.0, 45.4, 51.6, 60.7, 65.9, 72.3, 72.4, 128.4, 128.5, 129.1, 138.1, 156.3, 174.6; anal. calcd for C_19_H_27_NO_6_: C, 62.45; H, 7.45; N, 3.83; found: C, 62.19; H, 7.10; N, 4.13.

Ethyl (8R*,8aS*)-octahydroindolizine-8-carboxylate ((±)-5)


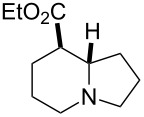


A yellow oil (*R*_f_ 0.55, CH_2_Cl_2_/MeOH 95:5); yield: 41%; ^1^H NMR (400 MHz, CDCl_3_) δ 1.27 (t, *J* = 7.15 Hz, 3H, CH_3_), 1.46–1.53 (m, 2H, CH_2_), 1.57–1.92 (m, 5H, CH_2_), 1.99–2.10 (m, 3H, CH_2_), 2.13–2.20 (m, 1H, CH_2_), 2.25–2.31 (m, 1H, H-8), 3.03–3.10 (m, 2H, CH_2_ and H-8a); ^13^C NMR (100 MHz, CDCl_3_) δ 14.6, 20.9, 25.1, 29.5, 30.1, 48.4, 52.6, 54.4, 60.6, 65.6, 174.6; MS (ESI) *m*/*z*: 198.5 [M + 1]; anal. calcd for C_11_H_19_NO_2_: C, 66.97; H, 9.71; N, 7.10; found: C, 66.60; H, 10.02; N, 7.39.

((8R*,8aS*)-Octahydroindolizin-8-yl)methanol; ((±)-tashiromine (±)-6) [35–37]


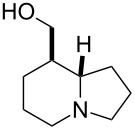


A yellow oil (*R*_f_ 0.45, CH_2_Cl_2_/MeOH/NH_4_OH 90:8:2); yield: 48%; ^1^H NMR (400 MHz, CDCl_3_) δ 1.00–1.11 (m, 1H, CH_2_), 1.42–1.53 (m, 2H, CH_2_), 1.58–1.83 (m, 5H, CH_2_), 1.84–1.99 (m, 3H, CH_2_), 2.04–2.11 (m, 1H, CH_2_), 3.03–3.10 (m, 2H, N-CH), 3.41–3.46 (m, 1H, OCH_2_), 3.59–3.64 (m, 1H, OCH_2_); ^13^C NMR (100 MHz, CDCl_3_) δ 21.1, 25.5, 28.0, 29.4, 44.9, 53.1, 54.5, 66.0, 66.9; MS (ESI) *m*/*z*: 156.6 [M + 1]; anal. calcd for C_9_H_17_NO: C, 69.63; H, 11.04; N, 9.02; found: C, 69.28; H, 10.70; N, 8.76.

Ethyl (1S*,2S*)-2-(benzyloxycarbonylamino)cyclooct-5-enecarboxylate ((±)-7)


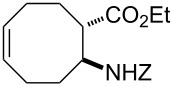


A colourless oil (*R*_f_ 0.55, *n*-hexane/EtOAc 4:1); yield: 48%; ^1^H NMR (400 MHz, DMSO) δ 1.18 (t, *J* = 7.10 Hz, 3H, CH_3_), 1.55–1.64 (m, 2H, CH_2_), 1.73–1.97 (m, 2H, CH_2_), 2.04–2.19 (m, 2H, CH_2_), 2.33–2.47 (m, 2H, CH_2_), 2.68–2.75 (m, 1H, H-1), 3.88–4.02 (m, 3H, OCH_2_ and H-2), 4.96–5.02 (m, 2H, OCH_2_), 5.53–5.60 (m, 2H, H-5 and H-6), 7.30–7.44 (m, 5H, Ar-H); ^13^C NMR (100 MHz, DMSO) δ 14.8, 24.5, 25.1, 29.2, 33.8, 49.2, 52.1, 60.4, 65.9, 128.0, 128.4, 129.0, 129.1, 130.7, 138.2, 156.9, 174.5; anal. calcd for C_19_H_25_NO4: C, 68.86; H, 7.60; N, 4.23; found: C, 68.57; H, 7.28; N, 3.97.

Ethyl (8S*,8aS*)-octahydroindolizine-8-carboxylate ((±)-9)


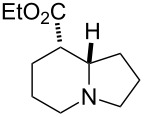


A yellow oil (*R*_f_ 0.45, CH_2_Cl_2_/MeOH 4:1); yield: 40%; ^1^H NMR (400 MHz, CDCl_3_) δ 1.28 (t, *J* = 7.15 Hz, 3H, CH_3_), 1.28–1.33 (m, 1H, CH_2_), 1.42–1.55 (m, 2H, CH_2_), 1.58–1.64 (m, 1H, CH_2_), 1.68–1.86 (m, 3H, CH_2_), 2.02–2.18 (m, 3H, CH_2_), 2.23–2.27 (m, 1H, CH_2_), 2.78–2.81 (m, 1H, H-8), 3.04–3.10 (m, 2H, CH_2_ and H-8a), 4.09–4.17 (m, 2H, OCH_2_); ^13^C NMR (100 MHz, DMSO) δ 15.1, 21.2, 22.6, 26.8, 27.3, 42.0, 53.4, 55.0, 60.5, 64.9, 170.4; MS (ESI) *m*/*z*: 198.7 [M + 1]; anal. calcd for C_11_H_19_NO_2_: C, 66.97; H, 9.71; N, 7.10; found: C, 67.28; H, 9.40; N, 6.78.

((8S*,8aS*)-Octahydroindolizin-8-yl)methanol; ((±)-epitashiromine, (±)-10) [32,34,39]


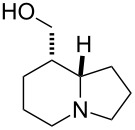


A yellow oil (*R*_f_ 0.45, CH_2_Cl_2_/MeOH/NH_4_OH 88:8:4); yield: 53%; ^1^H NMR (400 MHz, CDCl_3_) δ 1.56–1.62 (m, 2H, CH_2_), 1.65–1.74 (m, 4H, CH_2_), 1.97–2.12 (m, 5H, CH_2_), 2.20–2.28 (m, 1H, H-8), 2.94–3.00 (m, 1H, CH_2_), 3.08–3.14 (m, 1H, N-CH), 3.70–3.75 (m, 1H, OCH_2_), 4.13–4.19 (m, 1H, OCH_2_); ^13^C NMR (100 MHz, CDCl_3_) δ 21.2, 23.7, 26.3, 30.1, 35.7, 54.0, 54.9, 66.0, 66.8; MS (ESI) *m*/*z*: 156.4 [M + 1]; anal. calcd for C_9_H_17_NO: C, 69.63; H, 11.04; N 9.02; found: C, 69.30; H, 10.71; N, 8.79.

## References

[1] Michael J P (2008). Nat Prod Rep.

[2] Brandi A, Cardona F, Cicchi S, Cordero F M, Goti A (2009). Chem – Eur J.

[3] Macchi B, Minutolo A, Grelli S, Cardona F, Cordero F M, Mastino A, Brandi A (2010). Glycobiology.

[4] Felpin F X, Lebreton J (2003). Eur J Org Chem.

[5] Hu X-G, Hunter L (2013). Beilstein J Org Chem.

[6] Faria A R, Salvador E L, Correia C R D (2002). J Org Chem.

[7] Jiang X-P, Cheng Y, Shi G-F, Kang Z-M (2007). J Org Chem.

[8] Trajkovic M, Balanac V, Ferjancic Z, Saicic R N (2014). RSC Adv.

[9] Davies S G, Fletcher A M, Foster E M, Houlsby I T T, Roberts P M, Schofield T M, Thomson J E (2014). Org Biomol Chem.

[10] Rao N N, Parida B B, Cha J K (2014). Org Lett.

[11] Kim G-W, Jin T, Kim J-S, Park S-H, Lee K-H, Kim S-S, Myeong I-S, Ham W-H (2014). Tetrahedron: Asymmetry.

[12] Du-a-man S, Soorukram D, Kuhakarn C, Tuchinda P, Reutrakul V, Pohmakotr M (2014). Eur J Org Chem.

[13] Yoon H, Cho K S, Sim T (2014). Tetrahedron: Asymmetry.

[14] Kauloorkar S V, Jha V, Jogdand G, Kumar P (2014). Org Biomol Chem.

[15] Lingamurthy M, Rajander A, Rao B V (2014). Tetrahedron: Asymmetry.

[16] Ortiz G X, Kang B, Wang Q (2014). J Org Chem.

[17] Suga H, Hashimoto Y, Yasumura S, Takezawa R, Itoh K, Kakehi A (2013). J Org Chem.

[18] Hanessian S, Soma U, Dorich S, Deschênes-Simard B (2011). Org Lett.

[19] Han M-Y, Jia J-Y, Wang W (2014). Tetrahedron Lett.

[20] Singh P, Manna S K, Panda G (2014). Tetrahedron.

[21] Tiwari D K, Bharadwaj K C, Puranik V G, Tiwari D K (2014). Org Biomol Chem.

[22] Jouanno L-A, Di Mascio V, Tognetti V, Joubert L, Sabot C, Renard P-Y (2014). J Org Chem.

[23] McElhinney A D, Marsden S P (2005). Synlett.

[24] Marsden S P, McElhinney A D (2008). Beilstein J Org Chem.

[25] Bates R W, Boonsombat J (2001). J Chem Soc, Perkin Trans 1.

[26] Amorde S M, Jewett I T, Martin S F (2009). Tetrahedron.

[27] Chiou W-H, Lin Y-H, Chen G-T, Gao Y-K, Tseng Y-C, Kao C-L, Tsai J-C (2011). Chem Commun.

[28] Pohmakotr M, Prateeptongkum S, Chooprayoon S, Tuchinda P, Reutrakul V (2008). Tetrahedron.

[29] Bélanger G, Larouche-Gauthier R, Ménard F, Nantel M, Barabé F (2006). J Org Chem.

[30] Dieter R K, Watson R (2002). Tetrahedron Lett.

[31] Kim S-H, Kim S-I, Lai S, Cha J K (1999). J Org Chem.

[32] Dieter R K, Chen N, Watson R T (2005). Tetrahedron.

[33] David O, Blot J, Bellec C, Fargeau-Bellassoued M-C, Haviari G, Célérier J-P, Lhommet G, Gramain J-C, Gardette D (1999). J Org Chem.

[34] Thorat R G, Pansare S V (2013). Eur J Org Chem.

[35] Cutter A C, Miller I R, Keily J F, Bellingham R K, Light M E, Brown R C D (2011). Org Lett.

[36] Gage J L, Branchaud B P (1997). Tetrahedron Lett.

[37] Conrad J C, Kong J, Laforteza B N, MacMillan D W C (2009). J Am Chem Soc.

[38] Reddy K K S, Rao B V, Raju S S (2011). Tetrahedron: Asymmetry.

[39] Banwell M G, Beck D A S, Smith J A (2004). Org Biomol Chem.

[40] Kazi B, Kiss L, Forró E, Fülöp F (2010). Tetrahedron Lett.

[41] Kiss L, Kazi B, Forró E, Fülöp F (2008). Tetrahedron Lett.

[42] Kiss L, Cherepanova M, Forró E, Fülöp F (2013). Chem – Eur J.

[43] Cherepanova M, Kiss L, Fülöp F (2014). Tetrahedron.

[44] Cherepanova M, Kiss L, Forró E, Fülöp F (2014). Eur J Org Chem.

[45] Brambilla M, Davies S G, Fletcher A M, Roberts P M, Thomson J E (2014). Tetrahedron.

[46] Lee M-r, Stahl S S, Gellman S H (2008). Org Lett.

[47] Fricke Y, Kopp N, Wünsch B (2010). Synthesis.

[48] Malik G, Guinchard X, Crich D (2012). Org Lett.

[49] Sousa C A D, Rizzo-Aguiar F, Vale M L C, García-Mera X, Caamaño O, Rodríguez-Borges J E (2012). Tetrahedron Lett.

[50] Pérez-Bautista J A, Sosa-Rivadeneyra M, Quintero L, Höpfl H, Tejeda-Dominguez F A, Sartillo-Piscil F (2009). Tetrahedron Lett.

[51] Caputo F, Cattaneo C, Clerici F, Gelmi M L, Pellegrino S (2006). J Org Chem.

[52] Robinson A, Thomas G L, Spandl R J, Welch M, Spring D R (2008). Org Biomol Chem.

[53] Forró E, Árva J, Fülöp F (2001). Tetrahedron: Asymmetry.

[54] Palkó M, Benedek G, Forró E, Wéber E, Hänninen M, Sillanpää R (2010). Tetrahedron: Asymmetry.

